# Prognostic analysis and association of the systemic immune-inflammatory index with immune checkpoint inhibitor pneumonitis in patients with non-small cell lung cancer

**DOI:** 10.3389/fonc.2025.1596223

**Published:** 2025-06-12

**Authors:** Mengyao Cai, Jiangqian Sun, Jingyi Wu, Ya Liu, Yuanyi Huang

**Affiliations:** Department of Radiology, Jingzhou Hospital Affiliated to Yangtze University, Jingzhou, China

**Keywords:** non-small-cell lung cancer, checkpoint inhibitor-associated pneumonitis, systemic immune-inflammatory index, neutrophil-to-lymphocyte ratio, severity

## Abstract

**Objective:**

The Systemic Immune-Inflammatory Index (SII) is a comprehensive indicator reflecting immune response and disease burden. However, its significance in immune checkpoint inhibitor-related pneumonitis (CIP) in cases of non-small cell lung cancer (NSCLC) remains poorly explored. This study evaluated the association between SII and the incidence, severity, and prognostic effects of CIP in NSCLC patients.

**Methods:**

A retrospective analysis involved 215 NSCLC patients receiving immune checkpoint inhibitor (ICI) therapy, of whom 35 developed CIP while 180 did not. Baseline clinical characteristics and dynamic changes in peripheral blood biochemical markers were analyzed. Risk factors associated with the onset and severity of CIP were assessed, along with the diagnostic application of the SII for CIP.

**Results:**

Multivariate logistic regression identified smoking history (odds ratio [OR]: 3.23; *p* = 0.01), pre-existing lung disease (OR: 3.36; *p* < 0.01), squamous cell carcinoma (OR: 2.39; *p* = 0.03), and combined ICI therapy (OR: 4.77; *p* < 0.01) as independent risk factors for CIP onset. SII was also identified as independently predictive of severe CIP (OR: 6.35; *p* = 0.04). Receiver operating characteristic (ROC) curves demonstrated that SII had moderate accuracy for diagnosing CIP (area under the curve [AUC]: 0.63) and high diagnostic accuracy for severe CIP (AUC: 0.81). Multivariate Cox regression also showed that severe CIP was substantially related to reduced overall survival (OS) relative to mild CIP (hazard ratio [HR]: 0.06, 95% confidence interval [CI]: 0.01–0.52; *p* = 0.01).

**Conclusion:**

The results suggested the potential of SII as an indicator for diagnosing the presence and severity of CIP. Elevated SII levels were independently associated with the development of severe CIP, which, in turn, emerged as a key prognostic factor influencing overall survival in affected patients.

## 1 Introduction

Globally, lung cancer is a leading cause of cancer-associated mortality, with non-small cell lung cancer (NSCLC) accounting for > 80% of diagnosed cases (1, 2). Recently, therapeutic strategies for NSCLC have advanced considerably, offering a broad spectrum of anticancer interventions such as surgical resection, chemotherapy, radiation therapy, targeted agents, and immunotherapeutic approaches (3). The application of immune checkpoint inhibitors (ICIs) has significantly transformed the oncology field, establishing itself as a frontline treatment modality for various solid cancers, including NSCLC (4). Although ICIs demonstrate remarkable efficacy in targeting malignant cells, they may also disrupt immune homeostasis in normal tissues, resulting in various immune-related adverse events (irAEs) that can involve multiple organ systems (5). Among these, immune checkpoint inhibitor-related pneumonitis (CIP) is an uncommon but potentially fatal complication (6). The overall occurrence and fatality rate of CIP is estimated to be around 10-17%, while real-world data indicate that its prevalence among NSCLC patients varies between 4.8 and 39.3% (7).

The diagnosis of CIP relies on thoroughly ruling out other potential causes, including infectious pneumonia, tumor progression, lymphangitic carcinomatosis, pulmonary edema, thromboembolic disease, and radiation-induced pneumonitis, conditions generally unrelated to the pharmacological effects or radiological features of immunotherapy (8). The diagnostic approach to CIP typically integrates an evaluation of clinical symptoms, thoracic imaging findings, and microbiological analyses (sputum, blood, and urine cultures). Bronchoalveolar lavage fluid (BALF) is among the most commonly used invasive diagnostic procedures for CIP, with evidence indicating that specific lymphocytic profiles in BALF are associated with both disease onset and severity (9, 10).

Although lung biopsy is infrequently performed for CIP diagnosis, limited cases involving bronchial biopsies have demonstrated inflammatory changes and lymphocytic infiltration within pulmonary tissue after ICI therapy (11). Still, invasive procedures such as BALF analysis or lung biopsy are not routinely performed in all patients with suspected CIP.

Hematological markers of inflammation, which indicate the body’s inflammatory response, are readily available, cost-effective, and highly reproducible, making them valuable tools in CIP diagnosis and cancer prognosis. Among these biomarkers, the neutrophil-to-lymphocyte ratio (NLR) has been extensively investigated, as neutrophilia and lymphopenia are strongly related to the severity of systemic inflammation (12). Besides, systemic immune-inflammatory index (SII), initially proposed by Hu et al. (13) as a prognostic inflammatory biomarker for postoperative outcomes in hepatocellular carcinoma patients undergoing radical resection, is calculated by combining peripheral blood platelet count (PLT), lymphocyte count (LYM), and neutrophil count (NEUT). This index comprehensively evaluates the hemostasis between disease severity and immune function, highlighting better prognostic value than other inflammatory markers. SII has been widely recognized for its efficacy in predicting the onset and progression of various conditions, including cardiovascular disorders, COVID-19, pancreatitis, community-acquired pneumonia, etc (14–17). A similar study reported a significant association between elevated SII levels and clinical outcomes in patients with NSCLC undergoing treatment with ICIs (18). Yet, limited studies have explored the association between SII and the incidence of CIP and its prognostic implications in NSCLC patients.

Accordingly, this study evaluated the association between SII and CIP incidence and severity while also assessing the diagnosis of SII in predicting CIP occurrence and its prognostic significance in affected patients.

## 2 Methods

### 2.1 Ethical approval

This retrospective study was conducted at Jingzhou Hospital, a Yangtze University, China subsidiary. Ethical approval for the study was obtained from the respective Ethics Committee (approval #: 2024-134-01). As the research involved secondary data analysis and using pre-existing medical records and biological samples, the Ethics Committee waived the requirement for informed consent.

### 2.2 Study population and criteria

Patients diagnosed with stage III or IV NSCLC who received at least one course of immune checkpoint inhibitor (ICI) therapy between January 2021 and December 2023 were retrospectively included in the study. Exclusion criteria included patients with pre-existing drug-induced pneumonitis caused by drugs other than ICIs before immunotherapy and those with lung infections (e.g., tuberculosis, bacterial infections, fungal infections).

### 2.3 Diagnosis of CIP and assessment of pre-existing pulmonary disease

The diagnostic criteria for CIP comprised newly emerging infiltrative changes on chest imaging and/or new/worsening respiratory symptoms (e.g., dyspnea, cough, wheezing) after ICI treatment, with other causes (e.g., infection, tumor progression, pulmonary embolism, edema) excluded. In cases of suspected CIP, a comprehensive diagnostic evaluation was undertaken, incorporating lab tests (e.g., hematological analysis, tumor markers, D-dimer measurement, blood gas analysis, calcitonin), sputum cultures, and bronchoalveolar lavage. In patients with a history of radiation therapy, radiation-induced pneumonitis was ruled out by comparing the radiation field with the distribution of pulmonary infiltrates. Definitive diagnosis was established through transbronchial lung biopsy or percutaneous needle biopsy when initial evaluations were inconclusive and the patient’s clinical status allowed for such invasive procedures.

In this study, pre-existing lung disease was defined to include interstitial lung disease (ILD) and chronic obstructive pulmonary disease (COPD). ILD was diagnosed based on the presence of bilateral ground-glass opacities, reticular shadows, nonemphysematous cysts, or honeycombing on chest imaging. COPD was defined either by a pre-bronchodilator forced expiratory volume in 1 sec (FEV_1_) to forced vital capacity (FVC) < 0.70, or by the presence of clinical symptoms (chronic cough, sputum production, exertional dyspnea) in combination with extensive emphysematous changes observed on chest imaging.

### 2.4 Data collection

Clinical baseline data were retrospectively extracted from the medical record system, including age, sex, body mass index (BMI), smoking history, radiation therapy, pre-existing pulmonary disease, tumor histology, clinical stage, and treatment-related details. For CIP patients, additional variables were documented, including the duration of CIP, severity (graded by CTCAE v4.0: grades 1-2 = mild, ≥ 3 = severe), and clinical outcomes. The onset of CIP represented the interval between the administration of the initial ICI dose and the date of CIP diagnosis. The overall survival (OS) was observed from the date of CIP diagnosis to either the time of death or the end of follow-up (June 30, 2024).

Peripheral blood parameters were obtained at two distinct time points: at baseline (within one week before initiating ICI therapy) and at the time of CIP diagnosis (within 24 h before diagnosis) for CIP patients. For patients without CIP, blood samples were collected initially (baseline) and within one week before recent ICI administration. Evaluations included NEUT, LYM, PLT counts, and albumin (ALB) and hemoglobin (HB) levels. The NLR was calculated as NEUT/LYM, while SII was measured as (NEUT × PLT)/LYM.

### 2.5 Data analysis

Data were analyzed *via* SPSS 25.0 (IBM Corp., USA). Continuous variables were shown as either mean ± standard deviation (SD) or median with interquartile range (IQR), as per the data distribution. Independent samples t-tests or Mann-Whitney U tests were applied for variable comparisons based on normality and variance assumptions. Categorical variables were reported as frequencies (n) and percentages and were examined *via* chi-square tests.

Univariate and multivariate logistic regression analyses recognized distinct risk factors for CIP development. Peripheral blood biochemical markers with temporal variations were examined using the paired t-test or nonparametric rank-sum test. For parameters with considerable temporal changes, receiver operating characteristic (ROC) curves were used to assess the diagnostic performance of peripheral blood values at the time of CIP diagnosis and the most recent ICI treatment, along with NLR and SII at CIP diagnosis, in predicting CIP occurrence and severity. One-way and multivariate logistic regression were also performed, using the median value at CIP diagnosis as the threshold, to detect possible biomarkers related to CIP and chronic CIP.

Furthermore, the Cox proportional hazards model evaluated prognostic risk factors for CIP, while patient survival was detected *via* the Kaplan-Meier (K-M) method. Variations between groups were observed *via* log-rank tests. A two-sided *p*-value was used in all statistical analyses, with *p* < 0.05 indicating significance.

## 3 Results

### 3.1 Participants

Among the 257 patients diagnosed with NSCLC treated with ICIs, approximately 215 met the study criteria (inclusion and exclusion criteria) and were enrolled. All participants were divided into two groups as per the presence or absence of CIP, with 180 patients showing no signs of CIP and 35 patients diagnosed with CIP.

The median age of the 35 patients with CIP was 63 years (IQR: 59-67), with a higher proportion of males, about 88.6% (31/35). The median interval between initial ICI treatment and the diagnosis of CIP was 115 days (IQR: 86 - 172). Fourteen of the patients with CIP had severe CIP (40.0%) (**Table 1**).

**Table 1:** Baseline characteristics of CIP group and non-CIP group.

Variables	Non-CIP group (n=180)	CIP group(n=35)	P-value
Age			
Median(ranges)	64 (55–69)	63 (59–67)	0.66
<65, n (%)	102 (56.7)	21 (60.0)	0.85
≥65, n (%)	78 (43.3)	14 (40.0)	
BMI (kg/m^2^)	23.18 ± 2.87	23.27 ± 2.83	0.86
Gender, n (%)			
Male	146 (81.1)	31 (88.6)	0.34
Female	34 (18.9)	4 (11.4)	
Smoking status, n (%)			0.01
Never	82 (45.6)	7 (20.0)	
Current/former	98 (54.4)	28 (80.0)	
Preexisting lung disease, n (%)			<0.01
COPD/ILD	52 (28.9)	21 (60.0)	
No	128 (71.1)	14 (40.0)	
Prior radiation, n (%)			0.24
Yes	57 (31.7)	15 (42.9)	
No	123 (68.3)	20 (57.1)	
Histologic type, n (%)			<0.05
Squamous	74 (41.1)	21 (60.0)	
Non-squamous	106 (58.9)	14 (20.0)	
Pathological stage, n (%)			0.45
III	80 (44.4)	18 (51.4)	
IV	100 (55.6)	17 (45.6)	
Treatment line, n (%)			0.68
1	131 (72.2)	27 (77.1)	
≥2	49 (27.2)	8 (22.9)	
Treatment data, n (%)			0.01
Monotherapy	66 (36.7)	5 (14.3)	
Combination therapy	114 (63.3)	30 (85.7)	

CIP, checkpoint inhibitor-related pneumonitis; BMI, body mass index; COPD, chronic obstructive pulmonary disease; ILD, interstitial lung disease.

In univariate and multivariate logistic regression analyses, smoking history (odds ratio [OR]: 3.23, 95% confidence interval [CI]:1.27-8.21; *p* = 0.01), underlying lung disease (OR:3.36,95% CI:1.51-7.49; *p* < 0.01), squamous carcinoma (OR:2.39,95% CI:1.08-5.30; *p* = 0.03) and ICI combination therapy (OR:4.77,95%CI:1.66-13.67; *p* < 0.01) were independently predictive of CIP development (**Table 2**).

**Table 2:** Logistic regression analysis of the CIP group and the non-CIP group.

Variables	Univariate analysis		Multivariate analysis	
	OR (95%CI)	P-value	OR (95%CI)	P-value
Age (≥65 vs. <65)	0.85 (0.41-1.78)	0.67		
Gender (male vs. female)	1.81 (0.60-5.46)	0.30		
Smoking (current or former vs. never)	3.35 (1.39-8.06)	<0.01	3.23 (1.27-8.21)	0.01
Preexisting lung disease	3.69 (1.74-7.81)	<0.01	3.36 (1.51-7.49)	<0.01
Prior radiation	1.62 (0.77-3.39)	0.20		
Histology (squamous vs. non-squamous)	2.15 (1.03-4.50)	0.04	2.39 (1.08-5.30)	0.03
Pathological stage (IV vs. III)	0.76 (0.37-1.56)	0.45		
Treatment line (≥2nd vs. 1st)	0.79 (0.34-1.86)	0.59		
Treatment (combination vs. monotherapy)	3.47 (1.29-9.39)	0.01	4.77 (1.66-13.67)	<0.01

OR, odds ratio; CI, confidence interval.

### 3.2 Correlation of SII with CIP occurrence

**Figure 1:**
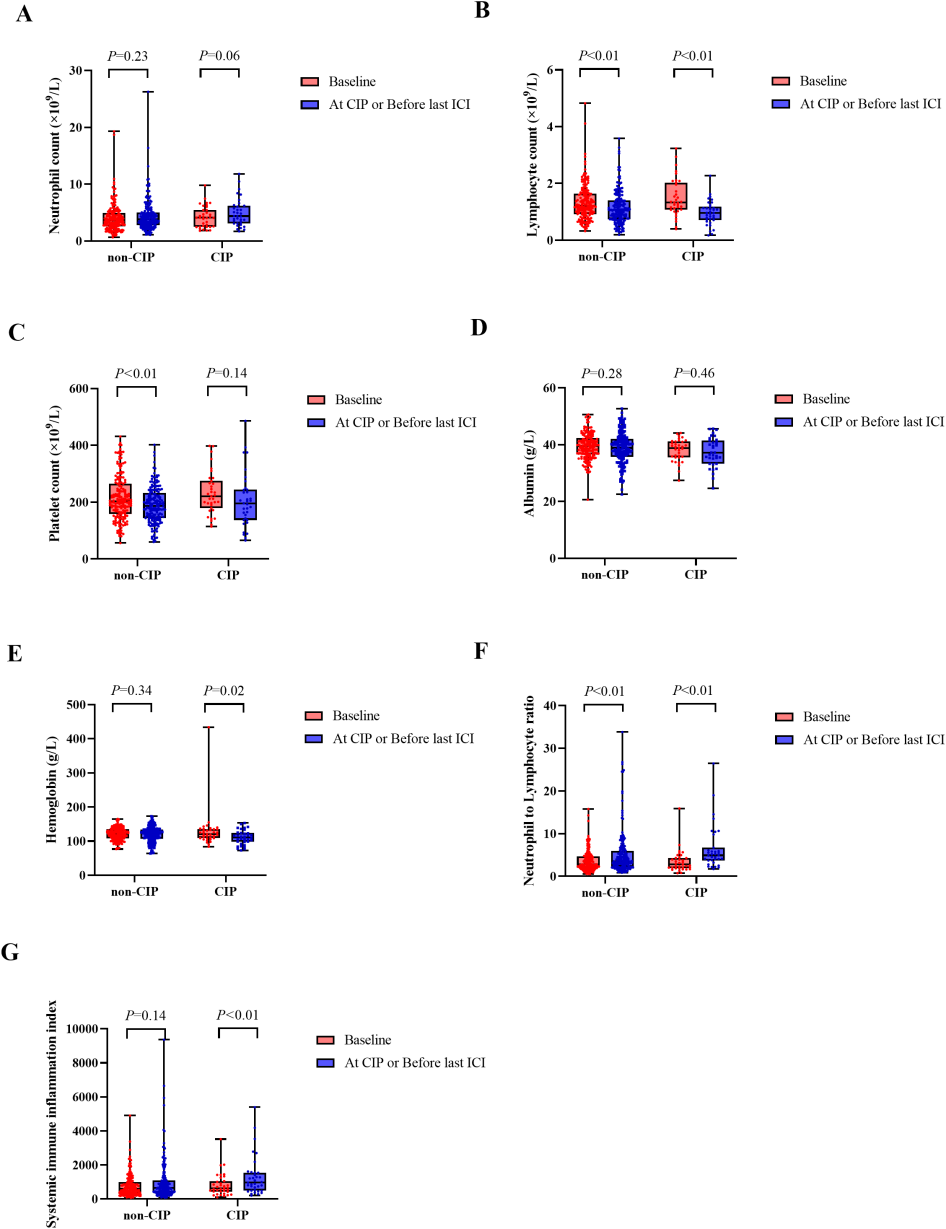
Box plot of peripheral blood parameters in patients with CIP and those without CIP at different time points. **(A)** Neutrophil counts. **(B)** Lymphocyte counts. **(C)** Platelet counts. **(D)** Albumin levels. **(E)** Hemoglobin levels. **(F)** Neutrophil-to-lymphocyte ratio. **(G)** Systemic immune inflammation index. CIP, checkpoint inhibitor-related pneumonitis; ICI, immune checkpoint inhibitors.

When CIP occurred, LYM reduced considerably from a baseline level of 1.33 × 10^9^/L (IQR: 1.07-2.03) to 0.96 × 10^9^/L (IQR: 0.71-1.18; *p* < 0.01), and similarly, in the non-CIP group, LYM reduced substantially from the baseline level to the most recent dose of ICI before treatment [1.21 × 10^9^/L (IQR: 0.92-1.64) to 1.06 × 10^9^/L (IQR: 0.74-1.40; *p* < 0.01)] (**Figure 1B**). In the non-CIP group, PLT reduced considerably from baseline to the most recent dose of ICI before treatment [202.95 × 10^9^/L (IQR: 158.25-264.50) to 144.00 × 10^9^/L (IQR: 186.95-232.00; *p* < 0.01)], but in the CIP group, it did not change over time [221.00 × 10^9^/L (IQR: 179.00-275.00) to 196.00×10^9^/L (IQR: 137.00-244.00; *p* = 0.14)] (**Figure 1C**). In the CIP group, there was a remarkable reduction in hemoglobin (HB) from baseline to CIP diagnosis [121.00 g/L (IQR: 109.00-135.00) to 110.00 g/L (IQR: 98.00-124.00; *p* = 0.02)]. Nonetheless, in the non-CIP group, it did not change over time [122.00 g/L (IQR: 108.00-134.75) to 122.80 g/L (IQR: 107.00-134.00; *p* = 0.34)] (**Figure 1E**).

When CIP occurred, NLR elevated substantially from a baseline level of 2.78 (IQR: 1.89-4.32) to 4.91 (IQR: 3.71-6.71, *p* < 0.01), and similarly, NLR in the non-CIP group increased substantially from baseline levels to the most recent dose of ICI before treatment [2.77 (IQR: 1.99-4.67) to 3.52 (IQR: 2.19-5.97; *p* < 0.01)] (**Figure 1F**). In the CIP group, a remarkable elevation in SII was observed from baseline to the time of CIP diagnosis, increasing from 629.91 (IQR: 433.13–1051.07) to 961.93 (IQR: 495.15–1545.11; *p* < 0.01). Yet, in the non-CIP group, SII failed to change over time [605.33 (IQR: 346.17-995.02) to 653.21 (IQR: 381.73-1086.73; *p* =0.14)] (**Figure 1G**).

### 3.3 Diagnostic value of the SII in CIP occurrence

**Figure 2:**
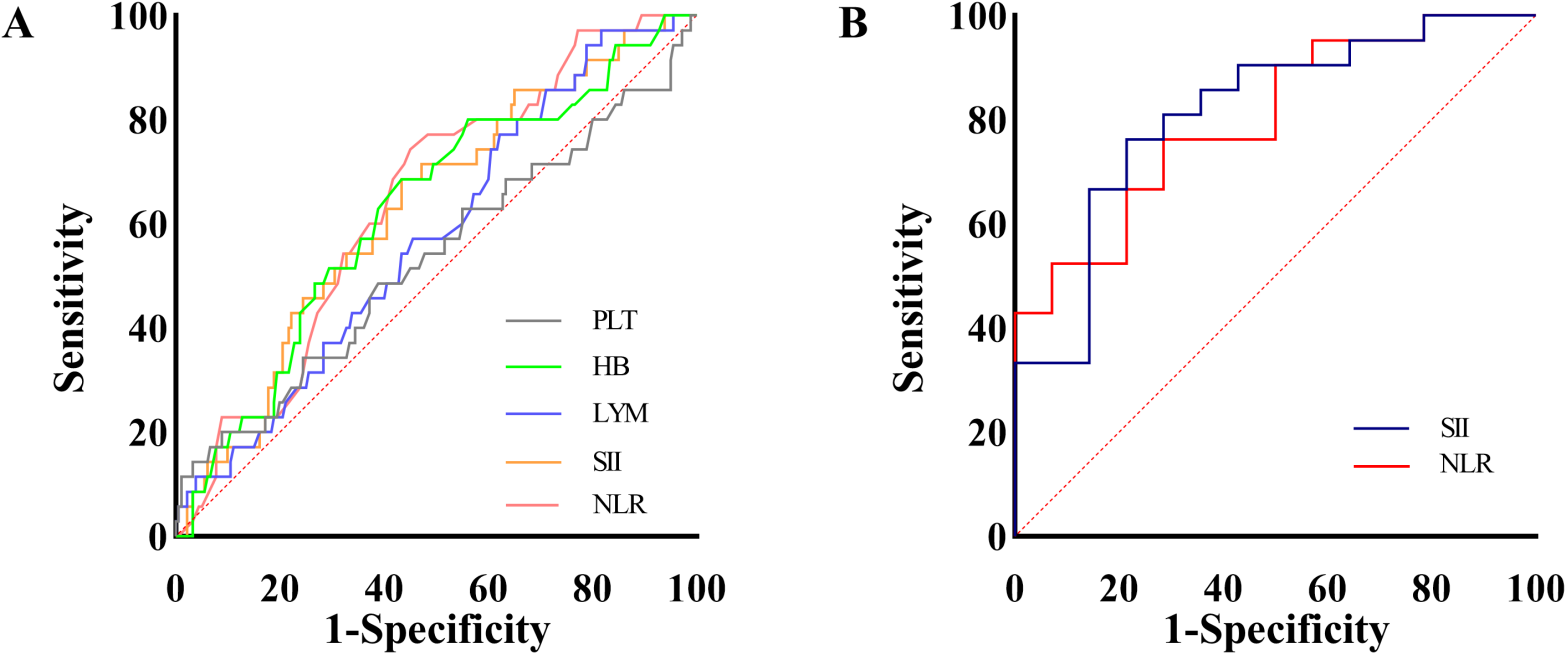
ROC curves for CIP occurrence **(A)** and severity **(B)**. LYM, Lymphocyte count; PLT, Platelet count; the units for LYM and PLT are both 1 ×10^9^/L; HB, Hemoglobin; expressed as g/L; NLR, Neutrophil-to-lymphocyte ratio; SII, Systemic immune inflammation index; CIP, checkpoint inhibitor-related pneumonitis; ROC, receiver operating characteristic.

To evaluate the diagnostic efficacy of changes in peripheral blood parameters, ROC curves were plotted based on data obtained at the time of CIP diagnosis and immediately after the most recent treatment of ICI. The optimal cutoffs were: HB 118.5 g/L (sensitivity 69%, specificity 57%, area under the curve [AUC] 0.63); NLR 4.04 (sensitivity 71%, specificity 58%, AUC 0.64); and SII 746.10 (sensitivity 69%, specificity 57%, AUC 0.63). PLT and LYM had AUC > 0.5 but were not significantly associated with CIP occurrence (*p* > 0.05) (**Table 3**; **Figure 2A**).

**Table 3:** Diagnostic efficacy of peripheral blood biochemistry for the development of CIP.

Variables	Cut-off	AUC	95%CI	Sensitivity	Specificity	P-value
LYM	1.63	0.58	0.48-0.68	0.97	0.18	0.16
HB	118.50	0.63	0.53-0.73	0.69	0.57	0.02
PLT	273.5	0.53	0.42-0.65	0.20	0.91	0.56
NLR	4.04	0.64	0.55-0.73	0.71	0.58	<0.01
SII	746.10	0.63	0.53-0.73	0.69	0.57	0.02

LYM, Lymphocyte count; PLT, Platelet count; the units for LYM and PLT are both ×10^9^/L; ALB, Albumin, expressed as g/L; HB, Hemoglobin, expressed as g/L; NLR, Neutrophil-to-lymphocyte ratio; SII, Systemic immune inflammation index; AUC, area under the curve; CI, confidence interval.

### 3.4 Correlation of SII with severe CIP

Among individuals diagnosed with CIP, 14 cases (40.0%) progressed to severe CIP. Univariate logistic regression analysis demonstrated a positive association between increased NLR, SII, and CIP severity levels. Multivariate logistic regression further identified an elevated SII level as a risk factor for severe CIP (OR: 6.35, 95% CI: 1.09-37.21; *p* = 0.04) (**Table 4**).

**Table 4:** Logistic regression analysis of the mild group and the severe group.

Variables	Univariate analysis		Multivariate analysis	
	OR (95%CI)	P-value	OR (95%CI)	P-value
NEUT (>4.38 vs. ≤4.38)	1.79 (0.45-6.97)	0.41		
LYM (≤0.96 vs. >0.96)	2.40 (0.60-9.67)	0.22		
PLT (>196 vs. ≤196)	2.93 (0.72-11.91)	0.13		
ALB (>37.2 vs. ≤37.2)	0.91 (0.24-3.52)	0.89		
HB (≤110 vs. >110)	1.47 (0.38-5.72)	0.58		
NLR (>4.91 vs. ≤4.91)	5.00 (1.14-21.80)	0.03	2.14 (0.38-12.25)	0.39
SII (>961.93 vs. ≤961.93)	9.17 (1.87-44.92)	<0.01	6.35 (1.09-37.21)	0.04

NEUT, Neutrophil count; LYM, Lymphocyte count; PLT, Platelet count; the units for NEUT, LYM, and PLT are ×10^9^/L; ALB, Albumin, expressed as g/L; HB, Hemoglobin, expressed as g/L; NLR, Neutrophil-to-lymphocyte ratio; SII, Systemic immune inflammation index; OR, odds ratio; CI, confidence interval.

### 3.5 Ancillary diagnostic value of SII in severe CIP

ROC curves were used to examine the diagnostic value of SII and NLR in identifying severe CIP. The optimal threshold for SII was 1047.51, yielding sensitivity 79% and specificity 76%. ROC curves further evaluated the diagnostic accuracy of both SII and NLR in predicting severe CIP. The optimal cutoff values and their diagnostic performance were as follows: SII: 1047.51 (sensitivity 79%, specificity 76%, AUC 0.81), NLR: 5.34 (sensitivity 71%, specificity 76%, AUC 0.80) (**Table 5**; **Figure 2B**). SII demonstrated slightly higher diagnostic efficacy for severe CIP compared to NLR.

**Table 5:** Diagnostic performance analysis of SIRI and NLR.

Variables	Cut-off	AUC	95%CI	Sensitivity	Specificity	P-value
NLR	5.34	0.80	0.65-0.94	0.71	0.76	<0.01
SII	1047.51	0.81	0.67-0.96	0.79	0.76	<0.01

NLR, Neutrophil-to-lymphocyte ratio; SII, Systemic immune inflammation index; AUC, area under the curve; CI, confidence interval.

### 3.6 Correlation between SII and overall survival

**Figure 3:**
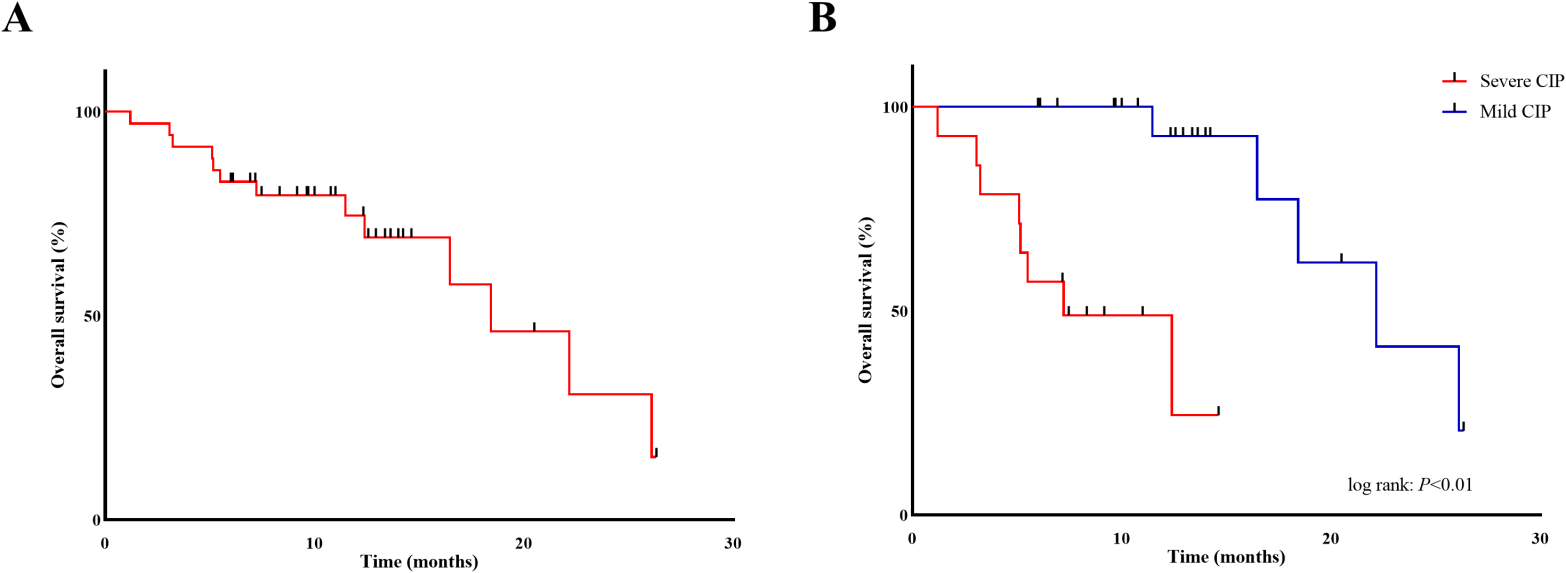
Kaplan-Meier curves of overall survival in patients with CIP **(A)** with stratification for CIP grade **(B)**. CIP, checkpoint inhibitor-related pneumonitis.

Among individuals who developed CIP after ICI treatment at Jingzhou Hospital of Changjiang University, 22 patients (62.9%) remained alive after the follow-up period on June 30, 2024, while 13 patients (37.1%) unfortunately died. The median OS for all CIP patients was 18.43 months (95% CI: 12.36–24.50 months), with a one-year survival rate of 74.6% (**Figure 3A**).

**Figure 4:**
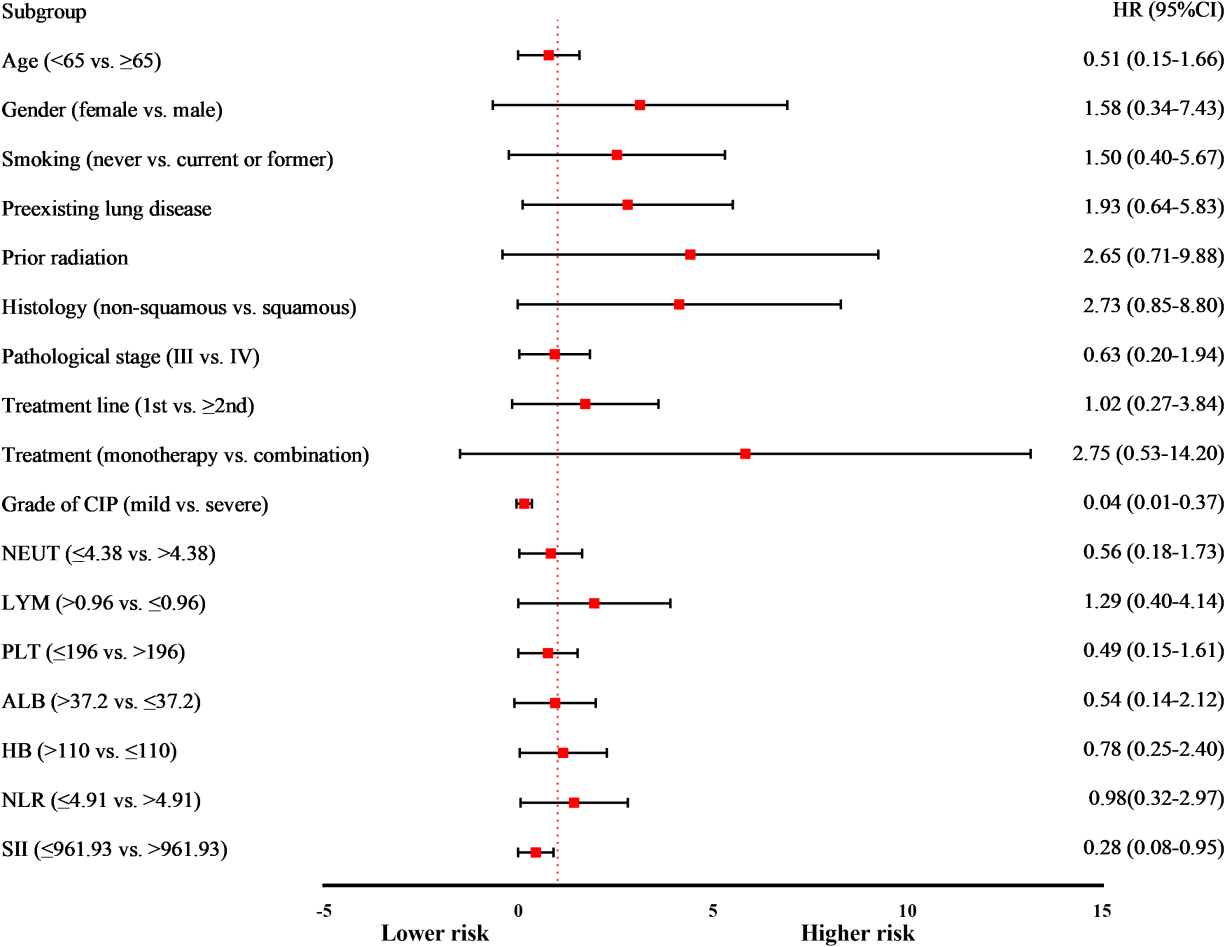
Forest plot of subgroup analyses of prognostic factors for overall survival in patients with CIP. CIP, checkpoint inhibitor-related pneumonitis; NEUT, Neutrophil count; LYM, Lymphocyte count; PLT, Platelet count; the units for NEUT, LYM, and PLT are 1 ×10^9^/L; ALB, Albumin, expressed as g/L; HB, Hemoglobin, expressed as g/L; NLR, Neutrophil-to-lymphocyte ratio; SII, Systemic immune inflammation index; HR, hazard ratio; CI, confidence interval.

A univariate Cox proportional hazards model was developed to analyze variables during CIP diagnosis. The findings indicated that CIP severity, SII levels, and OS were significantly correlated with CIP severity (mild vs. severe; hazard ratio [HR]: 0.04, 95% CI: 0.01- 0.37; *p* < 0.01) and SII levels (≤ 961.93 vs. > 961.93; HR: 0.28, 95% CI: 0.08–0.95; *p* = 0.04) (**Table 6**; **Figure 4**).

**Table 6:** Cox regression analysis of overall survival in CIP patients.

Variables	Univariate analysis		Multivariate analysis	
	HR (95%CI)	P-value	HR (95%CI)	P-value
Age (<65 vs. ≥65)	0.51 (0.15-1.66)	0.26		
Gender (female vs. male)	1.58 (0.34-7.43)	0.56		
Smoking (never vs. current or former)	1.50 (0.40-5.67)	0.55		
Preexisting lung disease	1.93 (0.64-5.83)	0.25		
Prior radiation	2.65 (0.71-9.88)	0.15		
Histology (non-squamous vs. squamous)	2.73 (0.85-8.80)	0.09		
Pathological stage (III vs. IV)	0.63 (0.20-1.94)	0.42		
Treatment line (1st vs. ≥2nd)	1.02 (0.27-3.84)	0.97		
Treatment (monotherapy vs. combination)	2.75 (0.53-14.20)	0.23		
Grade of CIP (mild vs. severe)	0.04 (0.01-0.37)	<0.01	0.06 (0.01-0.52)	0.01
NEUT (≤4.38 vs. >4.38)	0.56 (0.18-1.73)	0.32		
LYM (>0.96 vs. ≤0.96)	1.29 (0.40-4.14)	0.67		
PLT (≤196 vs. >196)	0.49 (0.15-1.61)	0.24		
ALB (>37.2 vs. ≤37.2)	0.54 (0.14-2.12)	0.38		
HB (>110 vs. ≤110)	0.78 (0.25-2.40)	0.67		
NLR (≤4.91 vs. >4.91)	0.98(0.32-2.97)	0.97		
SII (≤961.93 vs. >961.93)	0.28 (0.08-0.95)	0.04	0.67 (0.16-2.81)	0.59

CIP, checkpoint inhibitor-related pneumonitis; NEUT, Neutrophil count; LYM, Lymphocyte count; PLT, Platelet count; the units for NEUT, LYM, and PLT are ×10^9^/L; ALB, Albumin, expressed as g/L; HB, Hemoglobin, expressed as g/L; NLR, Neutrophil-to-lymphocyte ratio; SII, Systemic immune inflammation index; HR, hazard ratio; CI, confidence interval.

In the multivariate Cox proportional hazards model, only CIP severity (mild vs. severe; HR: 0.06, 95% CI: 0.01–0.52; *p* = 0.01) showed a significant and independent association with OS in CIP patients (**Table 6**). The median OS was estimated at 22.17 months (95% CI: 14.76–29.58) for mild CIP and 7.23 months (95% CI: 1.59–12.87) for severe CIP, with significant variances between both groups as determined by the log-rank test (*p* < 0.05) (**Figure 3B**).

## 4 Discussion

The median time to the onset of CIP among NSCLC patients treated with ICIs was 115 days (IQR: 86 - 172), with an observed incidence rate of 16.3% (35/215 cases). A previous study has documented a higher incidence of CIP in real-world clinical settings compared to controlled clinical trials, a trend that aligns with the findings of the present study (19). This discrepancy may be attributed to increased clinical awareness and vigilance in detecting CIP and the potentially higher-risk profile of the patient population receiving immunotherapy.

A meta-analysis determined that the risk of CIP is lower in females compared to males (20), potentially due to the higher prevalence of smoking history among males. Chronic exposure to tobacco smoke can result in pulmonary damage and contribute to the development of chronic respiratory diseases, which may increase susceptibility to CIP. Several retrospective clinical studies have also identified a significant association between smoking history and CIP occurrence, with smoking status recognized as one of the most influential and independent prognostic factors in CIP development (21, 22). Previous research has indicated that squamous carcinoma may serve as a potential risk factor for CIP, likely due to its predominant occurrence as a central lung carcinoma, which increases the possibility of obstructive pneumonia and elevates the risk of CIP. A study by Chao et al. also identified COPD as a crucial risk factor for the development of CIP in NSCLC patients (OR: 3.21; 95% CI: 1.26–9.53). The microenvironment of inflammatory response in COPD patients differs markedly from that of those without the condition, as the persistent recruitment and activation of various T-cell subsets contribute to chronic pulmonary inflammation. This heightened immune activation within tumor-affected and normal lung tissues is likely to be closely involved in CIP pathogenesis (23). Multiple retrospective studies have demonstrated that patients with pre-existing ILD have a significantly higher incidence of CIP. Baseline chest imaging findings of fibrosis are the strongest independent predictor of CIP and are associated with a poorer prognosis. Fibrosis denotes a pathological process characterized by aberrant repair and remodeling of lung tissue. ICI therapy, by enhancing immune activation, promotes the progression of pulmonary fibrosis, thus perpetuating a deleterious cycle of inflammation and fibrotic remodeling, which increases the risk of CIP (24, 25). A meta-analysis of clinical trials investigating ICIs reported an overall CIP incidence of 1.6% after monotherapy and 6.6% after combination therapy, indicating a substantially elevated risk associated with combination regimens (26). These findings align with current evidence identifying squamous carcinoma, smoking history, pre-existing lung disease, and combination therapy as independent risk factors contributing to CIP development. Patients with characteristics such as male sex, a history of smoking (past or current), pre-existing lung disease, and increased susceptibility to CIP after combination immune therapy require increased clinical monitoring. In individuals presenting with these risk factors, the administration of ICIs necessitates a cautious approach, complemented by post-treatment monitoring to facilitate the prompt identification and appropriate management of immune-related adverse events.

Anti-PD-1 antibodies can induce abnormal immune cell activation, leading to cytotoxic attacks on various cell types, including type II alveolar and airway epithelial cells and vascular endothelial cells. This immune-mediated response triggers systemic inflammation and increases circulating inflammatory cells within the peripheral blood (27). Lymphocytes are pivotal in tumor immunosurveillance and immune response (28). Inhibition of ICI promotes the reactivation of depleted lymphocytes, restoring their antitumor activity. A reduction in LYM count is a frequently observed phenomenon during immunotherapy. LYM levels substantially reduced from baseline in both CIP and non-CIP groups, with significant variations (*p* < 0.05). On the other hand, this reduction showed limited diagnostic value in identifying CIP.

HB plays a vital role in oxygen transport, and its deficiency is associated with systemic hypoxia, which may contribute to compromised pulmonary function and heightened vulnerability to pneumonia. Lower HB levels are associated with weakened immune responses, leading to deficiencies in cellular immunity that can further facilitate pneumonia development (29). A study by Liu et al. (30) demonstrated that reduced HB levels in NSCLC patients before immunotherapy are associated with a high risk of CIP. AUC for pre-treatment HB levels in estimating CIP occurrence was 0.68 (95% CI: 0.60-0.76, *p* < 0.01), with a pre-treatment HB threshold of 120.0 (95% CI: 0.60-0.76, *p* < 0.01). The AUC of the pretreatment HB level in estimating the occurrence of CIP was 0.68 (95% CI: 0.60-0.76, *p* < 0.01), and the highest predictive value was found when the pretreatment HB value was 120.90 g/L, with a sensitivity of 68% and a specificity of 61%. In this study, lower HB levels were significantly observed at the onset of CIP, with values decreasing from a baseline of 121.00 g/L (IQR: 109.00-135.00) to 110.00 g/L (IQR: 98.00-124.00; *p* < 0.05). This significant reduction was not detected among individuals in the non-CIP group. Moreover, ROC curve analysis for CIP occurrence demonstrated an AUC of 0.62 (95% CI: 0.52-0.72, *p* < 0.05). The optimal predictive threshold for HB was 117.50 g/L, yielding sensitivity 69% and specificity 56%.

The SII combined NEUT, LYM, and PLT, providing a more comprehensive assessment of inflammatory and immune dynamics within the body compared to individual biomarkers (31). Elevated SII levels result from an increase in NEUT and PLT counts and a reduction in LYM levels. In this study, the difference between the increase in NEUT and PLT was not statistically significant, and the increase in SII was due to lymphocytopenia. This pattern contrasts with that of bacterial pneumonia, which is typically characterized by a predominant neutrophilic response. The predictive value of the SII for severe CIP remains relatively underexplored. Similar studies have identified SII as a reliable indicator for severe childhood Mycoplasma pneumonia, demonstrating high diagnostic performance compared to other inflammatory markers, i.e., the NLR, platelet-to-lymphocyte ratio, and systemic inflammatory response index (sensitivity = 0.876, specificity = 0.987, AUC = 0.940) (32). In the current analysis, SII emerged as a distinct risk factor for severe CIP, showing slightly higher diagnostic accuracy than NLR. Findings by Huai et al. indicated that elevated SII levels correlated with reduced OS after neoadjuvant immunotherapy in patients diagnosed with NSCLC (33). Furthermore, a meta-analysis revealed that SII values > 750 served as a prognostic marker for unfavorable OS outcomes in cancer patients undergoing ICI therapy (HR: 2.20, 95% CI: 2.04-2.82) (34). Multiple pathophysiological mechanisms may underlie the association between elevated SII and adverse survival outcomes. First, neutrophils contribute to tumor progression *via* direct interactions with malignant cells or by modifying the tumor microenvironment (35). Second, platelets facilitate cancer-related inflammatory responses by mediating the recruitment of immune and hematopoietic cells to tumor sites (36). Lastly, lymphocytes, as key mediators of cellular immunity, play a crucial role in antitumor responses (37). In the present analysis, only univariate findings indicated that elevated SII levels during CIP diagnosis correlated with reduced OS in affected patients.

Previous studies have demonstrated that patients experiencing irAEs show a substantial prolonged OS relative to those without irAEs (38). However, alternative findings indicate that while grade 1–2 CIP is associated with a more favorable prognosis, this correlation does not extend to grade 3–4 CIP cases (39). In this study, the overall death rate among patients diagnosed with CIP was 37.1%. Further analysis demonstrated a substantially higher mortality rate in the severe CIP group (57.1%, 8/14) relative to the mild CIP group (23.8%, 5/21). The multivariate Cox proportional hazards model identified CIP severity as an independent prognostic factor, with an HR of 0.04 (95% CI: 0.01–0.45, *p* < 0.01), favoring mild over severe CIP. The median OS was 22.17 months (95% CI: 14.76–29.58) for patients with mild CIP and 7.23 months (95% CI: 1.59–12.87) for those with severe CIP. The difference in survival outcomes between the two groups was substantial, as indicated by the log-rank test (*p* < 0.05). Although mortality directly attributable to severe CIP is uncommon, CIP significantly impairs respiratory function, thus adversely influencing OS. Furthermore, the management of severe CIP often necessitates the permanent discontinuation of immunotherapy and/or the administration of high-dose corticosteroids, both of which may attenuate the antitumor efficacy of ICI and thus reduce the OS of the patients (40).

Several limitations are associated with the current study. Firstly, the diagnosis and severity assessment of CIP rely primarily on thoracic imaging and clinical symptomatology, without objective and specific biomarkers or standardized pathological criteria. Future studies should prioritize the identification of reliable biomarkers and the development of uniform pathological diagnostic frameworks to improve the accuracy and consistency of CIP diagnosis and severity stratification. Secondly, there was inconsistency in the timing of sample collection between the two groups, which may have introduced temporal bias. Future studies can increase statistical strength and help balance potential differences across various time points by expanding the sample size and conducting multicenter research. Lastly, this study was retrospective; thus, the study design needs to be further validated through prospective research.

## 5 Conclusion

This study concluded that the SII serves as a reliable diagnostic biomarker for the incidence and severity of CIP. Elevated SII levels represent a distinct risk factor for the occurrence of severe CIP. CIP severity is a key prognostic determinant, underscoring its crucial role in patient outcomes. These results highlight the clinical significance of SII in risk stratification and prognosis assessment in patients receiving ICI.

## Data Availability

The raw data supporting the conclusions of this article will be made available by the authors, without undue reservation.
